# Therapeutic Vaccination in Head and Neck Squamous Cell Carcinoma—A Review

**DOI:** 10.3390/vaccines11030634

**Published:** 2023-03-13

**Authors:** K. Devaraja, Sadhna Aggarwal, Manisha Singh

**Affiliations:** 1 Division of Head and Neck Surgery, Department of ORL-HNS, Kasturba Medical College, Manipal, Manipal Academy of Higher Education, Manipal, Udupi 576104, Karnataka, India; 2 Department of Radiation Oncology, The University of Texas MD Anderson Cancer Center, Houston, TX 77030, USA; 3 Department of Genomic Medicine, The University of Texas MD Anderson Cancer Center, Houston, TX 77030, USA

**Keywords:** head and neck cancer, squamous cell carcinoma, immunotherapy, vaccination, therapeutic vaccine, checkpoint inhibitors, human papilloma virus, dendritic cell, active-specific immune response

## Abstract

Therapeutic vaccination is one of the most effective immunotherapeutic approaches, second only to immune checkpoint inhibitors (ICIs), which have already been approved for clinical use. *Head and neck squamous cell carcinomas (HNSCC*s*)* are heterogenous epithelial tumors of the upper aerodigestive tract, and a significant proportion of these tumors tend to exhibit unfavorable therapeutic responses to the existing treatment options. Comprehending the immunopathology of these tumors and choosing an appropriate immunotherapeutic maneuver seems to be a promising avenue for solving this problem. The current review provides a detailed overview of the strategies, targets, and candidates for therapeutic vaccination in HNSCC. The classical principle of inducing a potent, *antigen-specific, cell-mediated cytotoxicity* targeting a specific *tumor antigen* seems to be the most effective mechanism of therapeutic vaccination, particularly against the *human papilloma virus positive* subset of HNSCC. However, approaches such as *countering the immunosuppressive tumor microenvironment* of HNSCC and *immune co-stimulatory mechanisms* have also been explored recently, with encouraging results.

## 1. Introduction

*Head and neck squamous cell carcinoma (HNSCC)* is a group of heterogenous tumors arising from the squamous epithelium of the upper aerodigestive tract that encompasses the oral cavity, pharynx, and larynx. According to a recent Global Cancer Statistics estimation, close to a million new cases of HNSCC were diagnosed globally in 2020, and approximately half a million of patients succumbed to HNSCC during that period [1]. Although a vast majority of these tumors are related to a few traditional risk factors, such as tobacco chewing and alcohol intake, there has been an appreciable shift in the epidemiology of HNSCC over the last few decades. A significant proportion of HNSCCs are now increasingly being linked to oncogenic strains of *human papilloma virus (HPV) infection*, such as HPV-16 and -18, particularly in developed nations. In 2015, The Cancer Genome Atlas (TCGA) published a comprehensive landscape of genomic alterations in HNSCC, providing further molecular insights into these two classes of HNSCCs, that has enabled greater advancement of the translational research towards optimizing the clinical outcomes in these groups [2]. With the emerging evidence over the last two decades, it has now become clear that HPV-driven HNSCCs exhibit peculiar biological behavior and fare better than HPV-negative and tobacco-related HNSCCs, correlating well with the differences in molecular characteristics of these two sub-sets of HNSCC [3,4]. Most notably, while the *loss of function of p53* and overexpression of *epidermal growth factor receptor (EGFR)* are the two major aberrations in HPV-negative HNSCC, the HPV-positive counterparts peculiarly exhibit wild-type p53 and do not overexpress EGFR. Instead, HPV-positive HNSCCs *overexpress the CDKN2A gene or p16 protein* and *amplify PIK3CA*, both of which are also prognostically favorable alterations [3]. Further, studies have also identified a relatively favorable *immune phenotype* in HPV-positive HNSCC, with higher scores of immune cells and a low mutation load as compared to the HPV-negative tumors [4,5].

Currently, the overall prognosis of HNSCC, with the existing treatment regimens, is unacceptably indigent. This sub-optimal therapeutic responsiveness is at least partly due to underlying molecular characteristics and immune phenotypes, which are mostly conducive of an immunosuppressing *tumor microenviroment (TME)*. Recently, a group of investigators analyzed the gene expression profiles of 944 HNSCC patients from 4 independent datasets and identified three distinct classes of HNSCC, each with a peculiar molecular and immune phenotype, as well as clinical responsiveness [6]. As per this classification, tumors belonging to cluster 1 exhibited the highest immune cell infiltration and had the best response to immunotherapy, and, thus, the best prognosis, while those in cluster 3 showed the lowest immune cell infiltration and therapeutic responsiveness and the worst prognosis. The results of this study reiterate the role of molecular characteristics, particularly of TME and *cytolytic T-lymphocyte (CTL)*, in imparting overall therapeutic responsiveness and prognosis in HNSCC [6].

In this regard, *immunotherapeutic modalities* seem to be a promising respite, as they not only tend to enhance the anti-tumor immunity of the host, but also may act by countering the immunosuppressive TME [7,8,9]. To date, pembrolizumab (KEYTRUDA, Merck and Co., Rahway, New Jersey, USA) and nivolumab (OPDIVO, Bristol Myers Squibb, New York, New York, USA), *two immune checkpoint inhibitors* (ICI), are the only immunotherapeutic medications to have received U.S. Food and Drug Administration (FDA) approval for use in HNSCC [7]. On the other hand, several recent developments in the field of immunotherapy have put *therapeutic vaccines* at the forefront of the immunotherapeutic armamentarium, and a few vaccination strategies have already come closer to obtaining a regulatory nod for their clinical use. The aim of this article is to provide a comprehensive overview of the various *approaches and apparatus of therapeutic vaccination in HNSCC*. Apart from introducing the immunopathological basis of therapeutic vaccination, this review also sheds light on the some of the promising vaccine candidates, as well as related trials and their outcomes.

## 2. Basic Principles of Therapeutic Vaccines

### 2.1. Mechanisms of Action

Conventionally, cell-mediated cytotoxicity, a phenomenon that hails from the concept of *immunosurveillance*, has been the central mechanism of the anti-tumor effect of therapeutic vaccines [10,11,12]. Historically, although the notion of an immune mechanism protecting the host from malignant tumors was prevalent among the scientific community for more than a century, it took several decades after the introduction of the first immunotherapeutic approach (in the form of Coley‘s toxin), for the experts to understand the actual mechanisms of *anti-tumor immunity*. In this regard, the proposal of the concept of *immunosurveillance* by Burnet and Thomas, and the subsequent key discoveries of 1960s, including that of T-lymphocytes, have enabled a significant advances in the field of translational research [11,12,13,14,15,16,17]. The *tumor antigens*, also known as *tumor rejection antigens*, are nothing but the protein/peptide sequences that are expressed largely, if not solely, by the precursor or the actual tumor cells. During the process of carcinogenesis, these antigens are generally released into the circulation. *Antigen-presenting cells (APC),* such as *dendritic cells (DC),* take up these *tumor antigens* from circulation and present them to host T cells, leading to their activation and differentiation into effector T cells and memory T cells. Subsequently, the reactivated T cells (CTL) carry out *tumor antigen*-specific cytolytic activity (*ASIR*) by releasing cytokines such as interferon (IFN)-γ/tumor necrosis factor (TNF)-α, perforin, and granzyme, which eradicate cancer cells [18,19]. The T cell responses also include secreting IL-2, which supports cellular immunity, and increasing the expression of CD80/86 costimulatory signals, which are essential for T cell priming [19].

Interestingly, shortly after the proposal of the *immunosurveillance* theory, immunologists discovered that the *tumor antigen-induced activation* of CTLs is also associated with the co-induction of a specific class of immunoglobulins that could counter the activity of CTLs [20,21]. Particularly, the identification of two prominent *immune checkpoints (IC),* the cytotoxic T-lymphocyte protein 4 (CTLA4) and programmed cell death protein 1 (PD-1), which attenuate the activity of T cells, attracted a great attention, and subsequently led to the introduction of a revolutionary group of immunotherapeutics, called *ICI* [22,23,24]. Some of the other prominent mechanisms that are known for hindering anti-tumor immunity are (a) abundance/activation of immunosuppressive cells, such as *tumor-associated macrophages (TAMs)*, *T regulatory cells (Tregs)*, and *myeloid-derived suppressor cells (MDSCs)*; (b) the secretion of immunosuppressive cytokines, such as *transforming growth factor-β (TGF-β)*; (c) the activation of immunosuppressive signaling pathways, such as *signal transducer and stimulator of transcription (STAT)-3*; and, last but not least, (d) the formation of physical barriers and intricate vascular networks within the tumor that hinder the drug delivery and render the tumor cells hypoxic [9]. A few other pro-oncogenic immunological factors that have also been identified in the recent times include the inhibition of chemotactic recruitment of APCs and effector cells (CTL), alterations to autophagy, resetting of telomerase, and cancer stem cell-mediated immunosuppression [25]. Nevertheless, all of these discoveries have now enabled researchers and oncologists to explore the mechanisms that could counter these anti-inflammatory responses as an alternative approach of delivering anti-tumor immunity [8,26,27]. Currently, a handful trials related to therapeutic vaccines are evaluating the strategies to convert *cold tumors* (tumors with predominately immunosuppressive TME) to *hot* (tumors with pro-inflammatory or anti-tumor TME), and the preliminary results of some of these trials have been encouraging so far [8,26,27].

To summarize the mechanism of actions, therapeutic vaccines aim at eradicating a tumor either by targeting a specific (or a group of) *tumor antigen*(s) to induce a robust ASIR against the tumor cells, or by countering the immunosuppressive factors in TME, that reactivates the naturally existing anti-tumor immunity. Some vaccines act by both of these mechanisms. While a vast majority of the therapeutic vaccines that have been under the radar for the last two decades work by delivering a *tumor antigen* or a derivate of one, that induces APC-presented and T cell mediated *ASIR* against the tumor cells, the last few years have seen a tremendous shift in translational research focusing on the principle of countering the immunosuppressive factors in TME [18].

### 2.2. Functional Units

#### 2.2.1. Tumor Antigens—Most Widely Used Targets of Therapeutic Vaccines

Conventionally, *tumor antigens* form the central functional unit for most of the therapeutic vaccines. These antigens can be *shared antigens (SHA),* expressed both in tumor cells and in normal tissues; *tumor associated antigens (TAA)*, upregulated in tumor cells but also weakly expressed in normal cells; or *tumor specific antigen (TSA)*, which are expressed only in tumor cells [28]. On the other hand, *tumor antigens* can also be categorized as per their peculiar expression characteristics [29]. Some of the relevant classes in this regard include *cancer/testis antigens (CTA)*, which are restricted to reproductive organs (testis and placenta) but are overexpressed by cancer cells; *oncoviral antigens*, encoded by tumorigenic viruses and, thus, found only on virus-infected tumor cells; *overexpressed/differentiation antigens*, which are found in normal tissues but are significantly overexpressed in cancer cells; and *mutated antigens* (also known as *neoantigens*), unique tumor antigens generated by a genetic mutation or alteration in transcription and found only in cancer cells [29,30,31]. Some of the prominent *tumor antigens* that have contributed greatly to the development of therapeutic vaccines, particularly in HNSCC, are the melanoma antigen-encoding gene (MAGE) (*CTA*), HPV-E6, E7 (*oncoviral*), Epstein–Barr virus (EBV)-related latent membrane protein (LMP)-2 (*oncoviral*), MUC-1, Wilm’s tumor (WT)-1, survivin, carcinoembryonic antigen (CEA) (*overexpressed and SHA*), and epidermal growth factor receptor(EGFR)-vIII *(neoantigen*) [32]. In order to facilitate further research on therapeutic vaccines, a working group of *the National Cancer Institute* (NCI) published a report in 2009, in which the *tumor antigens* were prioritized and ranked as per their clinical relevance [33]. However, after evaluating and weighting the 75 *tumor antigens*, the expert committee found that no single antigen could satisfy the criteria for an “Ideal” *tumor antigen*. Nevertheless, as per the relative priority, WT1 was the top-ranked *tumor antigen* in this pilot project, followed by MUC1, LMP2, HPV E6 E7, and EGFRvIII [33].

#### 2.2.2. Vehicles/Platforms of Vaccine Delivery

The *tumor antigens* or their epitopes (a part/peptide sequence of the antigens that can independently induce ASIR) can be delivered to the vaccine-recipient in several formats using a variety of vehicles. On one hand, the whole of the tumor cell, extracted from the patient and inactivated for oncogenicity in vitro, can be conjugated to a co-stimulating adjuvant and administered back to the same patient to induce a robust ASIR. On the other hand, an exogenous material that mimics a specific epitope of a *tumor antigen* can be injected directly to stimulate APC-mediated ASIR and tumor cell destruction.

Table 1 lists and discusses the characteristics of commonly used vaccine vehicles.

Being the most potent APC, DC is well known for its role in bridging innate and adaptive immunity, and thus has been a preferred vaccine vehicle for delivering either the whole tumor cell or the *tumor antigens* that could induce a potent ASIR. These vaccines are prepared by loading tumor antigens to patients’ autologous DCs ex vivo to be administered back to the same patients. Nevertheless, the limited availability of patient samples and the complexity involved in preparing the autologous vaccines led to a decrease in the popularity of DC-based vaccines. On the other hand, the recombinant vaccines, which are based on peptides from specific *tumor antigens* conjugated to adjuvants or immunomodulators, have gained wider acceptance. Currently, most of the peptide-based vaccines being evaluated in clinical trials target one of the CTAs, differentiation antigens, or oncofetal antigens. In addition, in most of these peptide vaccines, adjuvants such as *toll-like receptor* (TLR) are used to potentiate the otherwise modest immunogenicity of these TAAs [19]. The other noteworthy strategy to deliver a *tumor antigen* or epitope is to utilize viral vectors carrying expression cassettes. The first and most extensively evaluated viral-based therapeutic vaccine trials involved the poxviridae family, such as vaccinia, modified vaccinia strain Ankara (MVA), and the avipoxviruses (fowlpox and canarypox). Subsequently, recombinant adenovirus gained popularity owing to the ease of engineering and propagation for clinical use, as well as due to their ability to transduce both dividing and non-dividing cells for the high expression of transgenes [19].

#### 2.2.3. Vaccine Adjuvants

Finally, *adjuvants* play a crucial role in therapeutic vaccines, as they are likely to be involved, either directly or indirectly, in *tumor antigen* presentation to the host immune system and, thus, are known for enhancing the therapeutic efficacy of vaccination. It is no wonder that the DC, dubbed as *nature’s adjuvant*, is one of the most desired platforms for the development of vaccines [34,35,36]. Nevertheless, the realization of the efficacy-enhancing ability of this adjuvant has led to the introduction of several potent adjuvants over the years, such as *Montanide ISA (SEPPIC, Courbevoie, France)*, based on detoxified or *Freund’s incomplete adjuvant (IFA), granulocyte–macrophage colony-stimulating factor (GM-CSF), TLR-based adjuvants, polylactic acid, polylactide–coglycolide, and imiquimod* [37,38].

## 3. Approaches to and Apparatus of Therapeutic Vaccination in HNSCC

Of all the identified molecular alterations in HNSCCs, as per the NCI’s priority ranking of *tumor antigens* and the subsequently emerged literature, the most relevant antigens for the development of therapeutic vaccines are HPV E6/E7, EGFR, MAGE, p53, p16, WT1, MUC1, and LMP2 [2,6,33].

### 3.1. HPV Infection as the Target for Therapeutic Vaccines

**Figure 1:**
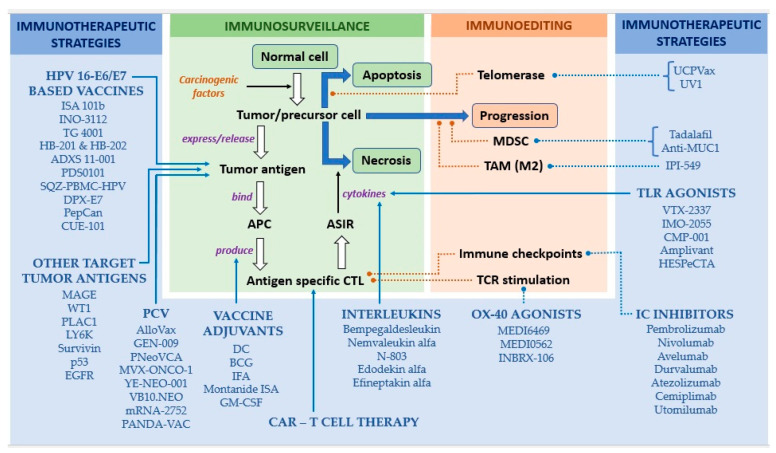
Immunological basis, approachs and agents of therapeutic vaccination in HNSCC. For abbreviations and further details, kindly refer to the Section 3 of text.

Transcriptionally active HPV viruses, being present only in tumor cells but not in normal cells, could serve as a perfect target for immune cells to effectively identify and kill HPV-positive tumor cells with minimal or no collateral damage. In fact, prophylactic HPV vaccines, such as Gardasil (Merck and Co., Rahway, NJ, USA) and Cervarix (GlaxoSmithKline Biologicals, Rixensart, Belgium), are the only vaccines, other than the vaccine for hepatitis B infection, to have received approval for the prevention of human cancers [39]. However, unlike Gardasil and Cervarix, which target the L1 capsid protein of the HPV virus to elicit the production of neutralizing antibodies that bind to the viral particles and block their entrance into host cells, the therapeutic vaccines cannot use the L1 capsid proteins, as these proteins are not expressed in infected basal epithelial cells. Thus, they will not aid in the identification or eradication of already-established infection [40]. On the other hand, early oncoproteins of HPV, such as E6 and E7, are not only expressed constitutively and at high levels in the infected cells but are also essential for the onset and maintenance of malignancy. Hence, they are unlikely to escape immune responses by mutation [3,40]. For these reasons, E6 and E7 proteins of high-risk HPV infection (HPV-16/-18 E6/E7) are the most-preferred *targets (as tumor antigens)* of the therapeutic vaccines, for the induction of ASIR against HPV-positive HNSCCs. E1 and E2 are other viral proteins of HPV, expressed at higher levels than E6 and E7 earlier in the process of carcinogenesis, and thus can be used for targeting HPV infection in its early stages [40]. These immunogenic antigens are usually integrated into a live vector or fused to a protein, a synthetic long peptide (SLP), or even nucleic acid. Several of these combinations are currently being explored for their safety and efficacy, either as monotherapies or in combination with other immunotherapeutic strategies, most commonly checkpoint blockade. Figure 1 illustrates the contemporary approaches and agents of therapeutic vaccination in HNSCC, and the basis of each of these approaches and their corresponding evidential statuses are summarized in Table 2.

#### 3.1.1. Vaccines Targeting HPV-16 E6/E7

##### ISI 101b

ISA 101 (ISA Pharmaceuticals, Leiden, Oegstgeest, The Netherlands) is an SLP-based vaccine containing 12 SLPs derived from the E6 and E7 oncogenic proteins of HPV-16. A single-arm, phase II clinical trial (NCT02426892) with 24 patients with incurable HPV-16-positive cancer (22 of 24 had oropharyngeal squamous cell carcinoma (OPSCC)), evaluated whether the vaccination with ISA101 would be able to amplify the efficacy of an approved anti-PD-1 antibody, nivolumab [41]. The overall response rate of 33% (36% OPSCC) and the median overall survival of 17.5 months that were reported in this study are promising compared to the prior reports with isolated anti-PD-1 inhibition. The recently published long-term results of this trial, after a median follow-up duration of 46.5 months (median survival duration of 15.3 months and a 3-year overall survival rate of 12.5%), are also encouraging [73]. In this study, the clinical responses correlated well with the CTL score found in the TME at the baseline (in pre-treatment biopsy specimens). Additionally, upon gene expression analysis of these specimens, a higher expression of genes related to immune response, and the IFN-signaling pathway also correlated significantly with the clinical response [73]. Currently, several other trials are evaluating the safety and efficacy of ISA 101 in combination with other ICI, and with different profiles of HPV-16+ OPSCC (NCT03669718, NCT04398524, NCT04369937). Considering the emerging evidence and expectations, recently, FDA has issued the designation of *Fast track* to ISA 101b for its use in recurrent/metastatic(R/M) HPV-16+ OPSCC [74].

##### INO-3112

INO-3112, (Inovio Pharmaceuticals Inc., Plymouth Meeting, PA, USA), a DNA-based vaccine, has two components; VGX-3100 a synthetic DNA plasmid targeting HPV-16 and -18 E6 and E7 antigens, and INO-9012, a DNA plasmid containing recombinant interleukin-12 (IL-12). A phase I/IIa study (NCT02163057) evaluated the safety, tolerability, and immunogenicity of INO-3112 in 22 HPV-16/-18 positive HNSCC patients between Aug 2014 and Jan 2017 [42]. The trial included two distinct groups of participants: patients in cohort I (n = 6) received immunotherapy before and after definitive surgery, while patients in cohort II (n = 16) received immunotherapy after the completion of concurrent chemoradiation. This study was carried out to demonstrate proof of tissue immune responses in paired tumor samples (cohort I) and to evaluate whether patients could mount an ASIR even after cisplatin-based concurrent chemoradiation therapy. There was no treatment-related grade 3–5 adverse event in any of the patients. Elevated antigen-specific T cell activity was noted in 18 of 21 evaluable patients, and persistent cellular responses were recorded out to one year. At the tissue level, a reversal or positive shift of the *CD8^+^/FoxP3^+^ ratio* (ratio of CTL:Treg) was observed after vaccination in four of five specimens evaluated. Interestingly, the number of perforin-positive immune infiltrates (used to determine the cytolytic capacity of the infiltrates) was increased in all five, suggesting a functional capability of antigen-specific CTL induced by the immunization [42]. However, two subsequent trials that were planning to evaluate the combination of INO-3112 and Durvalumab (Imfinzi, AstraZeneca, Cambridge, United Kingdom), a PD-L1 antibody, in patients with R/M heavily treated HPV-16/-18+ HNSCC (NCT03162224) and in the adjuvant setting of HPV-16+ OPSCC (NCT04001413), have been terminated (after partial accrual) and withdrawn, respectively. Interestingly, a phase II open-label study (NCT03439085) evaluating the efficacy of the same combination of INO-3112 and Durvalumab in patients with R/M HPV+ cancers of non-head and -neck regions since November 2018 has also stopped recruitment after reaching 77 participants. A detailed breakdown of the results of these trials might lead the direction of future research on INO-3112.

##### TG4001

TG4001 (Transgene, Illkirch-Graffenstaden, France) (also known as tipapkinogene sovacivec) is a non-propagative, highly attenuated vaccinia vector (MVA) which is engineered to express the coding sequences of the HPV16 E6 and E7 tumor-associated antigens and the cytokine, IL-2. A Phase Ib/II trial (NCT03260023) is currently evaluating the safety of a combination of TG4001 and Avelumab (BAVENCIO, Merck and Pfizer), an anti-PD-L1 agent, as well as the efficacy of this combination against avelumab alone, in patients with HPV-16+ R/M cancers, including HNSCC. A preliminary safety report on nine patients (five of whom had OPSCC) reported no dose-limiting toxicities or serious adverse events and confirmed a partial clinical response in three patients [43]. Interestingly, of the five evaluable patients, at day 43 of immunization, three showed a detectable E6/E7-specific T cell response in the periphery, and four demonstrated an increase in CD8 infiltration and/or a decrease in infiltrated Treg/CD8 ratio in tissue (positive shift of CTL:Treg ratio). There was also an increased expression of genes associated with both adaptive and innate immunity, shifting the tumor gene signature from a profile of *a cold tumor* to that of a *hot* tumor [43]. Both the phenotypic findings and the gene expression signatures suggest the induction of immune changes even in patients with poor baseline immune contexture [43].

##### HB-201 and HB-202

HB-201 and HB-202 are two live-attenuated vectors (TheraT^®^ Vectors, Hookipa Biotech GmbH, Vienna, Wien, Austria) based on the lymphocytic choriomeningitis virus and Pichinde virus, respectively, that express the same non-oncogenic HPV16 E7E6 fusion protein and infect APCs to induce tumor-specific T cell responses. A first-in-human phase I/II multinational, multicenter, open-label study (NCT04180215) started in Dec 2019 and is currently evaluating the safety, efficacy, immunogenicity, and clinical response of HB-201 and HB-202. In this study, HB-201 single vector therapy is being compared with HB-202/HB-201 alternating two-vector therapy for the recognition of an optimal/safe dosing schedule and therapeutic effectiveness in around 200 patients of R/M HPV 16+ cancers, including HNSCC. The study has two phases—phase I (dose escalation phase) to determine the recommended phase 2 dose (RP2D), and phase II (dose expansion phase) for further exploration, either alone or in combination with pembrolizumab. As per the preliminary analyses of the interim data from this ongoing trial, both HB-201 and HB-202/HB-201 are generally well tolerated and have been shown to rapidly induce high E6/E7-specific T cell levels in 65 heavily pre-treated patients with HPV16+ solid tumors (OPSCC being the most common site among the included patients) [44]. Interestingly, alternating two-vector therapy seems to maintain E6/E7-specific T cell responses more effectively by continuous dosing compared to single-vector therapy [75]. Currently, the identified RP2D is being tested with and without pembrolizumab in the study participants. In addition, in-depth sequencing of the paired biopsies is underway to characterize the TME in response to both single-vector and alternating two-vector therapy [44,75].

##### ADXS 11-001

ADXS 11-001 (Advaxis Inc., Princeton, NJ, USA) (also known as axalimogene filolisbac or AXAL) is a genetically-modified, nonpathogenic, live, attenuated Listeria monocytogenes listeriolysin O (Lm-LLO) engineered to secrete an HPV-E7 *tumor antigen* as a truncated LLO-E7 fusion protein. A window of opportunity trial (NCT02002182) is currently evaluating ADXS 11-001 LLO-E7 as a neoadjuvant vaccine in newly diagnosed HPV-positive OPSCC, prior to transoral robotic resection. An interim analysis involving eight recruited patients reported increased E6/E7-specific immune responses in the peripheral blood of five patients, as well as CD8 and CD4 intratumoral T cell infiltration in four patients [45]. In addition, the regulatory T cells were found to be decreased in three out of the six evaluable patients [45]. Although the estimated sample size of the trial was 30, it stopped recruitment recruiting after 15 participants and might publish the detailed analysis shortly. As per the update in ClinicalTrials.gov, there has been a measurable therapeutic response in the vaccinated group (n = 9) in the form of >2-fold increase in HPV-specific T cell response from the baseline to the time of surgery, and this increased further at three months post-surgery [76]. However, 55.5% of the vaccinated subjects seemed to have serious adverse events, as compared to 16.67% in the control group who did not receive vaccination prior to surgery (n = 6) [76]. These findings can be expanded upon only after the investigators publish detailed results and their interpretations.

Interestingly, a phase I/II trial (NCT02291055) that had planned to evaluate ADXS11-001, both alone and in combination with durvalumab, in a cohort of R/M HNSCC and cervical cancers seems to have suspended recruitment after enrolling 66 participants. As per a report, the trial was supposedly put on hold by the FDA for a few months, from March 9, 2018, to July 13, 2018, following the death of a patient that had occurred after the sixth cycle of combination therapy [77]. Similarly, another phase I dose-escalation trial (NCT01598792) on HPV-16+ OPSCC also observed *dose-limiting toxicity* in one of the two recruited patients with ADXS11-001, before being terminated due to withdrawal of support from manufacturers on commercial grounds. Similar results, i.e., a considerable degree of adverse events after vaccination, were also reported in trials related to cervical cancers [78,79].

##### PDS0101

PDS0101 (PDS Biotech, Florham Park, NJ, USA) is a liposomal-based HPV-16 E6/E7 multi-peptide vaccine containing immune-activating cationic lipid R-DOTAP, which is currently being evaluated in HPV+ HNSCC [80]. As a monotherapy, PDS0101 has shown efficacy against HPV+ tumors by generating HPV-specific T cells and anti-tumor activity in a mouse model. These activities were maximum when used with two immunomodulators, bintrafusp alfa and NHS-IL12, which are known to aid by countering the immunosuppressive TME. Bintrafusp alfa (also known as M7824) (Merck Serono, Darmstadt, Germany) is a first-in-class bifunctional fusion protein composed of the extracellular domains of the *TGF-β receptor type II* fused to a human IgG1 monoclonal antibody blocking PDL1, designed both as an *ICI* and to achieve the *TGFβRII ‘trap*’ TME. NHS-IL12 (Merck Serono, Darmstadt, Germany) is an immunocytokine designed to bring IL-12 to the TME [80,81]. These results led to a phase I/II clinical trial (NCT04287868) that is currently evaluating the safety and response rate of PDS0101 in combination with NHS-IL12 and bintrafusp alfa. In addition, an ongoing, multicentric phase II study (NCT04260126), called *VERSATILE002*, is evaluating the safety and efficacy of a combination of PDS0101 and pembrolizumab in checkpoint-naïve subjects with R/M/P HPV-16+ and PDL1+ HNSCC. Similarly, another ongoing, randomized, controlled phase I/II trial (NCT05232851) is evaluating the clinical benefits and survival outcomes of PDS0101, both as a monotherapy and in combination with pembrolizumab, in patients with LA HPV+ OPSCC. Recently published interim analyses of these two trials, with 15 and 18 enrolled patients, respectively, reported the combination to be safe and well-tolerated without any significant toxicity [82,83]. Moreover, the preliminary evidence suggests that the combination has a promising and durable clinical anti-tumor activity [83]. In fact, these reports have prompted the FDA to grant the *Fast track* designation to the combination of PDS0101 and pembrolizumab for use in R/M/P HPV16+ HNSCC [84].

##### SQZ-PBMC-HPV

SQZ-PBMC-HPV (SQZ Biotechnologies, Watertown, MA, USA) is a novel therapeutic cancer vaccine created with Cell Squeeze^®^, a proprietary cell-engineering system. After leukapheresis, the Cell Squeeze technology drives PBMCs through a microfluidic chip, leading to temporary cell membrane disruption and delivery of HPV16 E6 and E7 antigens cytosolically. SQZ-PBMC-HPV is neither genetically modified nor does it contain immune effector cells. A phase I multicenter study (NCT04084951) is currently evaluating the safety, tolerability, immunogenic effects, anti-tumor activity, and pharmacodynamics of SQZ-PBMC-HPV, both as a monotherapy and in combination with atezolizumab (Tecentriq, Hoffman La Roche Ltd., Basel, Switzerland) or any other ICI, in patients with LA or R/M HPV16+ solid tumors, including HNSCC. Interim results from 12 patients (3 HNSCC) demonstrated the clinical feasibility of the Cell Squeeze technology and the favorable tolerability of engineered APCs [46]. Additionally, the tumor analyses pre- and post-therapy indicated an increased immune response in some patients [46].

##### Other HPV-16 E7/E6 vaccines

Another vaccine based on the *HPV-16 E7aa 43-62* SLP has demonstrated anti-tumor activity when given intratumorally in a mouse model of oral squamous cell carcinoma (OSCC) [85]. The immune response induced by this vaccine was evident even in the absence of an adjuvant. Furthermore, the therapeutic effects found in this model were abolished upon the deletion of TLR-4, suggesting a role of the innate immune system in anti-tumor response, independent of CD4+ T cells [85]. Similarly, another novel vaccine, *HPV-16 E711-19 nanomer*, called *DPX-E7* (IMV Inc., Dartmouth, NS, Canada), is also being evaluated in HPV-16+ OPSCC (NCT02865135). The DPX-E7 has established its ability to induce ASIR in a mouse model [86]. In addition, compared to the untreated controls, the DPX-E7 immunized mice exhibited a lesser population of immunosuppressive cells in the TME [86]. A group of investigators from the University of Arkansas developed *PepCan*, a vaccine containing four synthetic peptides covering HPV16E6 and a novel adjuvant called candin, a colorless extract of *Candida albicans* [87,88]. They demonstrated the safety and therapeutic efficacy of *PepCan* against HPV infection-related warts and high-grade cervical intraepithelial neoplasia in phase I (NCT00569231) and phase II (NCT02481414) trials [88,89]. Recently, these investigators initiated a randomized, placebo-controlled, double-blinded phase I/II trial (NCT03821272) to evaluate the safety and efficacy of the *PepCan* vaccine regimen, which will be administered intradermally among the previously treated HNSCC patients who are in remission. Interestingly, like the previous studies by these investigators, this trial is also not taking HPV status into consideration. This is due to their goal of evaluating the ability of *PepCan* to reduce the recurrence rates regardless of HPV status.

*CUE-101* (CUE Biopharma, Boston, Massachusetts, USA), is an Fc fusion protein composed of four components-an Human leukocyte antigen (HLA) complex (HLA-A*0201), an HPV16 E7 peptide epitope (E7_11–20_), four molecules of reduced affinity human IL2 molecules and an effector-attenuated human IgG1 Fc domain [90]. It is the first Immuno-STAT to be investigated in HNSCC, and is designed to bind, expand, and activate HPV16-specific CD8+ T cells for the treatment of HPV16+ cancers [47]. It has demonstrated its ability to induce selective binding, activation and expansion of HPV16 E7_11-20_-specific CD8^+^ T cells in an animal model, and is currently being evaluated as a monotherapy and in combination with pembrolizumab in HPV16-positive HNSCC (NCT03978689). An interim analysis of this first-in-human trial demonstrated its safety and tolerability, with encouraging PD signals and anti-tumor activity in 53 enrolled patients [47].

#### 3.1.2. Other Targets Related to HPV Infection

##### p16_37-63

HPV-positive tumors are well-known to consistently overexpress the cyclin-dependent kinase inhibitor p16(INK4a), which can be used as a target for vaccination. A 27-amino-acid-long p16(INK4a)-based peptide vaccine, called p16_37-63 (Oryx GmbH & Co. KG, Baldham, Bayern, Germany) was evaluated in the VicOryx trial (NCT01462838). In this study, the p16_37-63 peptide vaccine, mixed with immunoadjuvant Montanide ISA-51 VG (IFA), was given to 26 eligible participants with previously treated R/M HPV-positive tumors at different sites. Of the participants, only 20 could receive at least four injections and were evaluable (7/20 had HNSCC). Although the immunization elicited a measurable immune response in 14/20 patients (7/7 with HNSCC) and led to clinically stable disease in nine of the 14 assessed patients (5/7 with HNSCC), this trial was discontinued prematurely, owing to progressive disease or death in the majority of the patients [48]. As an extension, VICORYX-2 (NCT02526316) also evaluated the combination of this p16_37-63 peptide vaccine and IFA with cisplatin-based chemotherapy in 11 treatment-naïve patients of LA HPV+ cancers, including HNSCC; however, the results are currently being awaited.

##### HARE-40

An ongoing, non-randomized phase I/II trial (NCT03418480) is evaluating the safety of the *HPV Anti-CD40 RNA Vaccine*, (BioNTech SE, Mainz, Germany), also known as HARE-40 in a cohort of HPV-driven SCC. In arm 1A of this study, 15 patients, previously treated for HPV16+ HNSCC who are currently disease-free, are scheduled to receive increasing doses of the HPV vaccine to establish a safe, tolerable, and recommended dosage. In arm 1B, 29 R/M HPV 16+ cancers (including HNSCC) are will be evaluated for a safe dosage in the palliative setting. This study was started in 2017, and is currently active and recruiting.

### 3.2. Other Virus-Related Immunotherapeutic Mechanisms in the Treatment of HNSCC

Although a detailed discussion of the various applications of different virus-based approaches in managing HNSCC is beyond the scope of this review, a few worthy additions to the discussion of HPV-based vaccination strategy are the vaccines targeting EBV in HNSCC, particularly in nasopharyngeal carcinomas (NPCs), and the role of oncolytic viruses in eradicating tumor cells. 

#### 3.2.1. Vaccination Strategies for NPC

The association between EBV infection and NPC is well-known, and as a result, several investigators are evaluating strategies to target EBV infection and related antigens/epitopes, for the purpose of inducing ASIR against the EBV+ NPC. The major *target-tumor antigens* of EBV are EBV nuclear antigen-1 (EBNA1) and LMP1/2 [91]. From the first attempts to create therapeutic vaccines by using EBV-primed DCs a few decades ago to the recent introduction of recombinant adenoviruses encoding for EBV-antigens, the vaccination strategies for management of NPC have progressed considerably [91]. A few not worthy approaches that are currently under evaluation include the use of virus-based vaccines and T-cell-based immunotherapies. A phase I trial (NCT01147991) has demonstrated the safety and immunogenicity of *MVA-EL*, a recombinant MVA-based EBNA1 C-terminal/LMP2 chimeric protein-expressing vaccine, in 16 patients with EBV + NPC [92]. A phase Ib/II trial (NCT03769467), which started in Feb 2019, identified a safe RP2D of a novel *allogenic, EBV-specific T-cell immunotherapy* agent, called *Tabelecleucel* (Atara Biotherapeutics, Thousand Oaks, CA, USA), in 12 patients with platinum-pretreated R/M EBV+ NPC (as a combination therapy with pembrolizumab) before being terminated by the sponsor. On the other hand, the status of a phase I trial (NCT04139057), evaluating the maximum tolerated dose (MTD) of EBV-specific T cell receptor (TCR)-T cells with anti-PD1 auto-secreted elements, in patients with EBV-positive HNSCC is currently unknown. Interestingly, another similar trial (NCT04509726) which is planning to evaluate the EBV-specific TCR-T cells with IL12 auto-secreted elements in a similar set of patients is yet to begin recruiting patients. Further detailed discussion on therapeutic vaccines for NPC is beyond the scope of this work, and can be found in a recent review [91].

#### 3.2.2. Oncolytic Viruses in HNSCC

Oncolytic virus therapy aims at destroying tumor cells by infecting them with a cytolytic virus. Such oncolytic viruses exhibit anti-tumor activity after the intratumoral injection(s), but may also affect metastatic tumors by inducing a systemic immune response. The most studied oncolytic viruses in HNSCC are *ONYX-015* (ONYX Pharmaceuticals, South San Francisco, CA, USA), *Talimogene laherparepvec,* also known as *T-VEC*, (Amgen Inc, Thousand Oaks, CA, USA), and *Pexastimogene Devacirepvec,* also known as, *Pexa Vec* or JX-594, (SillaJen Inc., Busan, Republic of Korea) [7]. ONYX-015 is an E1B-attenuated adenovirus engineered to selectively target, enter, and lyse p53-defective tumor cells. It was the first genetically engineered replication-competent virus to demonstrate selective intratumoral replication and necrosis in cancer patients [93]. It has demonstrated objective clinical responses with acceptable toxicity in phase II trials involving recurrent HNSCC [93,94]. *T-VEC* is an oncolytic herpes virus carrying GM-CSF, which has also shown tolerable safety as combination therapy with pembrolizumab in R/M HNSCC in a phase Ib/III trial (NCT02626000), called MASTERKEY-232 [95]. However, the efficacy of the combination was not different from that of pembrolizumab monotherapy in historical cohorts [95]. *Pexa-Vec* is a Wyeth-strain vaccinia oncolytic virus, engineered to express human GM-CSF, that positively impacts the immune system via several mechanisms, including activation of DCs and enhancement of CTL infiltration into tumors. A recent trial (NCT02977156) evaluating the safety and efficacy of intratumoral injection of *Pexa-Vec* in combination with ipilimumab (YERVOY, Transgene S.A., Illkirch-Graffenstaden, France) in LA or R/M solid tumors (including HNSCC) has completed its recruitment.

Lastly, there are a few reports of vaccination with live attenuated influenza virus being explored for its anti-tumor ability, particularly with the intention of converting the immunophenotypically *cold* tumors into *hot* [96,97,98]. However, there are no data regarding its feasibility or efficacy in HNSCC.

### 3.3. Non-Viral Tumor Antigens in HNSCC

#### 3.3.1. Whole Tumor Cells as a Source of Tumor Antigens

Intradermal injections of irradiated autologous tumor cells (ATC), admixed with BCG and vaccine-primed lymph node (VPLN) cells, have been shown to generate the appropriate immune response against the tumor cells in patients with advanced HNSCC [99]. Similarly, in a pilot study, 20 HNSCC patients were vaccinated with irradiated NDV-modified ATC vaccine three months after surgery [100]. The vaccination was safe and feasible, and the percentage of survival among the vaccinated patients five years later was 61%. Additionally, serial immune monitoring showed specific anti-tumor hypersensitivity and the presence of tumor-reactive T cells in the peripheral blood, even after five to seven years of vaccination in disease-free patients [100]. Interestingly, in order to overcome the tumor-induced immunosuppression and post-surgical/post-radiation reduction in T-lymphocytes, in both of these studies, investigators pre-conditioned the study participants with IL-2 prior to vaccination [99,101]. Further discussion regarding the role of IL-2 in rescuing the CTL can be found in subsequent textection, under the section on co-stimulatory vaccination strategies. A few recent trials have evaluated the role of DC in enhancing the anti-tumor activity of ATC. In a pilot trial, apoptotic ATC fused to DC were administered to patients of locally advanced-stage (LA) HNSCC who had been successfully treated with first-line therapy, but were at risk of recurrence or development of a second primary tumor. The serial immunological studies demonstrated the measurable immune response in vaccinated HNSCC patients (n = 4), and the generated responses targeted only the autologous tumor. Although the ATC-DC-based vaccine was safely tolerated in all those vaccinated, this study was terminated after five years due to overly stringent eligibility criteria and failure to enroll the proposed 12 patients [102]. On the other hand, in an animal model, the mice that received a combination of cancer stem cells (CSCs) fused to the DC vaccine and avasimibe (Pfizer, New York, USA), an acyl-CoA: cholesterol acyltransferase 1 (ACAT1) inhibitor, had relapsed tumors of smaller size and had longer mean survival time compared to those mice receiving either of these agents separately [103].

#### 3.3.2. CTA

Over 80% of the primary HNSCCs are known to express at least one CTA gene, and over 59% of the tumors could co-express three or more of such genes [104]. MAGE, the first immunogenic *tumor antigen* to have been discovered in humans, is also the most frequently over-expressed CTA in HNSCC [104].

##### MAGE

As per an estimate, more than half of HNSCC cases express the MAGE-A4 and MAGE-A3 genes [104]. In vitro stimulation with overlapping peptides encoding MAGE-A3 and MAGE-A4 was shown to induce specific CD4(+) T cells in all seven HNSCC patients evaluated [105]. A pilot study with Trojan vaccines, composed of HLA-I- and HLA-II-restricted MAGE-A3 or HPV-16 derived peptides, joined by furin-cleavable linkers and linked to a *penetrin* peptide sequence, showed the vaccine to have acceptable toxicity and good systemic immune responses against the HLA-II-restricted epitopes in five MAGE -A3/HPV 16+ patients of R/M HNSCC [106]. In this study, montanide ISA 51 and GM-CSF were used as adjuvants to promote DC migration to the site of vaccination and to enhance antigen presentation [106,107]. Later, a phase I trial (NCT00257738) with more cases of progressive/R/M HNSCC (HLA A2+) (nine HPV-16 positive and seven MAGE A3 positive) also reaffirmed the feasibility and safety of these vaccines [49]. The study, which was supposed to have enrolled 90 cases, could only enroll 17 patients (one of which turned out to be ineligible later on and was excluded) and, thus, was closed prematurely due to poor accrual. However, in both these studies, none of the immunized patients demonstrated any clinical response, either partial or complete.

A group of investigators found over-expression of MAGED4B in more than 50% of OSCC cases [108]. Later, they identified nine short peptides derived from the MAGED4B protein and demonstrated the immunogenicity and specificity of these peptide-pulsed DC-based vaccines against ex vivo OSCC [109]. Subsequently, these investigators evaluated the feasibility and immunogenicity of a dual-antigenic peptide vaccine comprising MAGED4B and another tumor antigen, four-jointed box 1 (FJX1), and found stronger immunogenic responses with the combination of these peptides than with any of those given individually [110]. Recently, they also evaluated this dual-antigenic vaccine globally and reported promising outcomes [111]. Interestingly, these vaccines not only inhibited the tumor growth and improved the clinical outcome, but also demonstrated an enhanced the clinical response in combination with anti-PD1 antibodies, resulting in tumor clearance in approximately 75% of mice [111].

##### WT1

WT1 is a well-known *tumor antigen*, overexpressed in various kinds of hematological malignancies and solid tumors, including HNSCC [112]. To reiterate, WT1 is the topmost *tumor antigen* as per the NCI pilot project ranking of tumor antigens [33]. Recently, a phase I/II trial (University Hospital Medical Information Network, Japan—000027279) on HNSCC used a WT1 peptide-loaded DC vaccine and OK-432 adjuvant in combination with conventional chemotherapy, and demonstrated its feasibility, safety, and reasonable clinical efficacy in patients of R/M HNSCC [113]. Interestingly, CUE-102 (CUE Biopharma, Boston, MA, USA), an Immuno-STAT, is almost similar to an HPV-16 E vaccine-CUE-101, except that it is based on a nine-amino-acid fragment of WT1 (WT1_37–45_) replacing the E7 peptide of CUE-101. This vaccine is currently under trial for various solid malignancies.

##### PLAC1

PLAC1 is an X-linked gene product known for its role in the development of placenta. Considering its expression in tumor tissues of different regions but not in normal tissue, it has been speculated to be involved in modulating tumor progression [114,115]. In a recent study, PLAC1 was highly expressed in 74.5% of oropharyngeal and 51.9% of OSCC specimens [116]. The investigators of this study identified a peptide epitope capable of inducing effective antigen-specific and tumor-reactive T cell responses. Furthermore, they found precursor T cells responding to PLAC1 peptide epitopes in the peripheral blood of HNSCC patients, suggesting a potential role of PLAC1 as a target antigen for immunotherapy, which requires further exploration [116].

##### LY6K

Tumor antigen *LY6K* is overexpressed in various tumors, including HNSCC, but the expression is undetectably low in normal cells [117]. Overexpression of LY6K is closely related to aggressive disease and poor prognosis, and is associated with recurrence and metastasis [118]. A long peptide vaccine derived from LY6K has demonstrated enhanced induction of LY6K-specific CTLs in HNSCC [119]. A phase II exploratory trial (UMIN-000008379) of a peptide-derived vaccine containing a mix of three CTAs (LY6K, CDCA1, and IMP3) and Montanide ISA51, in a cohort of 37 patients with LA or R/M HNSCC, reported a better clinical response and longer survival than the cohort (n = 18) that included HLA-A24 negative patients who were receiving best supportive care [120]. Interestingly, among the vaccinated patients, the enzyme-linked immunospot (ELISpot) assay (a highly sensitive immunoassay and the most commonly used method for quantitatively measuring the activity of antigen-specific T cells) identified LY6K-, CDCA1-, and IMP3-specific CTL responses in 85.7%, 64.3%, and 42.9% of the patients, respectively, and these antigen-specific responses correlated with the duration of overall survival [120].

#### 3.3.3. Other TAA in HNSCC

##### Survivin

*Survivin* is an *inhibitor of apoptosis proteins (IAP)*, abundantly expressed in most malignancies, but hardly detectable in normal adult tissues. The survivin-conjugated peptide has been shown to induce the production of survivin-specific CTL in patients with OSCC in vitro [121]. Further, a phase I trial (UMIN000000976) from the same center demonstrated the safety and reasonable therapeutic potential of survivin-2B peptide vaccination in HLA-A*2402 positive patients with unresectable, LA, or recurrent OSCC [122]. Although only one of the ten vaccinated patients demonstrated a clinical (partial) response, immunologically, a noticeable increase in the peptide-specific CTL was recorded in six of eight evaluated patients. However, in the opinion of the investigators, the amount of CTL induced by the vaccine was insufficient to result in tumor regression [122].

##### p53

p53, also called the *molecular policeman*, is a *tumor suppressor gene* that loses its function during the process of carcinogenesis in more than 80% of epithelial tumors, including HPV-negative HNSCC [3,123]. Although p53 can be utilized as a *tumor antigen* for therapeutic vaccines, in contrast to TSA, like MAGE, the p53 epitopes can be of two types—mutated (non-self) and non-mutated (wild-type p53) [124]. While the use of mutated p53 as a tumor antigen for therapeutic vaccines requires personalized preparations, limiting its practical feasibility, the wild-type p53 epitopes can be used effectively to induce the ASIR (similar to other TSAs), as most tumors with loss of function of p53 tend to overexpress p53 [123].

A phase I clinical trial (NCT00404339) that tested the tumor peptide-specific p53 vaccination (autologous DC loaded with wild-type p53) injected intranodally into inguinal nodes, has reported the strategy to be safe and effective, with promising clinical outcomes in 16 patients of advanced HNSCC who had received standard-of-care curative treatment [50,51]. The two-year disease-free survival (DFS) in this cohort was 88%, and the three-year DFS was 80%, which was better than the DFS (70%) seen in a similar cohort treated only with chemoradiation at the researchers’ institute [50]. Interestingly, the trial had planned to have 50 patients, but recruited only 17, and the results were only reported for 16 patients [51,125]. Another phase I study (NCT02432963) with 11 patients (one HNSCC) of p53-overexpressing solid cancers (defined as >10% of cells staining positive for p53) demonstrated that the vaccination with p53-expressing MVA (p53MVA), in combination of pembrolizumab, was effective, resulting in clinical benefits in a select few patients [52]. Although one patient in their cohort had a fatal myocarditis, no additional cardiac toxicities were noted after the amendment of study for enhanced cardiac monitoring. Last but not least, the loss of function of p53 could also be used as a target for oncolytic therapy with ONYX-15, as discussed earlier.

##### EGFR

HNSCC, particularly tobacco-related and HPV-negative HNSCC, are known for over-expression of EGFR, making it one of the attractive targets for therapeutic vaccines [3]. Vaccination with the DC, pulsed with recombinant glutathione-S-transferase (GST)-EGFR fusion protein, has been shown to induce a significant anti-tumor immune response against HNSCC, both in vitro and in vivo, in a mice model [126]. A phase I/II basket trial (NCT02955290) is currently studying the best dose and side effects of recombinant human EGF-rP64K/Montanide ISA 51 vaccine (CIMAvax) in combination with nivolumab in patients with metastatic non-small cell lung cancer or HNSCC. This trial, started in Dec 2016, is currently recruiting, and is estimated to enroll 193 patients.

### 3.4. Countering the Immunosuppressive TME of HNSCC

From the immunopathology point of view, the majority of HNSCCs tend to exhibit an *immune-depleted* phenotype upon histology [127,128]. Some of the *immune escape* mechanisms of HNSCC include the recruitment of inhibitory cell populations such as Treg, MDSC, TAM, and CAF; perturbation by ICs such as PD-1 and CTLA-4, leading to T cell exhaustion; dysregulation of pro-proliferative cytokines such as TGF-beta, IL-6, and IL-10, as well as signaling pathways such as STAT-3; and increased physical barriers that hinder the infiltration of effector T cells and other immune cells [129,130,131]. Interestingly, several of these immunological aberrations can be targeted and reprogrammed by therapeutic vaccines, thus enhancing anti-tumor immunity. The following sections discuss a few of the major approaches of therapeutic vaccines that act by reprogramming the TME to eradicate tumor cells in patients with HNSCC.

#### 3.4.1. Suppression of MDSC

MDSC, one of the predominant cells in *immune-depleted* tumors, not only provide immune protection to the tumor cells, but also regulate tumor angiogenesis and metastasis [55]. On the other hand, the elimination or inhibition of MDSC has been shown to restore CD8+ T cell activity in some of the tumors, including HNSCC [132,133]. In a mouse model and in in vitro experiments on cell lines of multiple myeloma and HNSCC, the use of the phosphodiesterase-5 (PDE5) inhibitor demonstrated the down-regulation of MDSC-suppressive pathways and the consequent restoration of anti-tumor immunity [133]. In fact, the role of PDE5 inhibition in augmenting both the general and tumor-specific immune response in HNSCC was further confirmed by two randomized, double-blinded, placebo-controlled clinical trials (NCT00894413 and NCT00843635) that used tadalafil for PDE5 inhibition [53,54]. In these trials, tadalafil was preferred over other PDE5 inhibitors, such as sildenafil and vardenafil, owing to the former’s better safety profile and long-acting PDE5 blockade with once-daily dosing. Another phase II trial (NCT01697800) evaluated tadalafil in conjunction with conventional therapy in 40 patients with HNSCC between Sept 2012 and July 2014, but has not published its results. As per the ClinicalTrails.gov page for the trial, of the 25 patients in the tadalafil group, one had mortality compared to none of 15 in the placebo group. Nevertheless, inspired by the results of preclinical studies on mouse tumor models and a few clinical trials on tadalafil, a randomized phase I/II clinical trial (NCT02544880) was initiated by the same investigators in April 2016 to evaluate whether tadalafil treatment increased the efficacy of another anti-tumor vaccine, Anti-MUC1, in patients with resectable (minimal residual) recurrent or second primary HNSCC [55]. A preliminary analysis of data from 14 patients reported the acceptable safety profile and encouraging immunologic potential of PDE5 inhibition in HNSCC [55]. Another reason for designing this study as combination therapy was the lack of dramatic clinical benefit with tadalafil monotherapy in earlier studies, despite the positive enhancement of anti-tumor immunity.

#### 3.4.2. Anti-MUC1 Vaccine

MUC is a family of high O-glycosylated proteins expressed only on the apical surfaces of luminal and glandular normal epithelial cells. Aberrant MUC expression is seen in several cancers, including HNSCC. Underglycosylated MUC1 is found in most HNSCC specimens, but usually remains undetectable in normal tissue [55]. A recent meta-analysis found a strong association between elevated MUC expression and detrimental clinicopathological outcomes [134]. Interestingly, as mentioned in the previous paragraph, an interim analysis of an ongoing trial (NCT02544880), of anti-MUC1 vaccine with tadalafil, reported the combination to be well-tolerated in 14 eligible HNSCC patients of the 16 enrolled [55]. It also demonstrated immunological evidence for positive immunomodulation and reversion of immune exclusion, suggesting an active role of tadalafil and an adjuvant to the anti-MUC1 vaccine in advanced HNSCC. Interestingly, the study also points to PDL1 as an additional mechanism of tumor evasion, adding support to the rationale for combining ICI and PDE5 inhibitors for the treatment of HNSCC. As per ClinicalTrials.gov, the trial has closed recruitment, and the detailed results are expected to be revealed soon.

#### 3.4.3. Remodeling the TAM

Macrophages, which play a key role in immunity, are primarily of two phenotypes. M1 is proinflammatory and secretes classic inflammatory cytokines that kill tumors by promoting tumor cell necrosis and immune cell infiltration into the TME. In contrast, M2 is anti-inflammatory, and exhibits powerful tumor-promoting functions, including degradation of the tumor extracellular matrix, destruction of the basement membrane, promotion of angiogenesis, and recruitment of immunosuppressor cells [135]. Although the TAM phenotype is mostly of M2 polarity, in general, TAM can be conditioned to transition between M1 and M2 [135,136]. While IFN-γ can stimulate macrophage phenotype switching from M2 to M1, aiding in anti-tumor immunity, the IL-4 polarizes TAM to M2 [137]. Remodeling the TME by reversing the TAM phenotype (transforming M2-polarization into M1-phenotype) is an important approach to anti-tumor immunotherapy, including therapeutic vaccines [135,136]. In this regard, selective pharmacologic targeting of the gamma isoform of phosphoinositide 3-kinase (PI3Kγ), which is highly expressed in myeloid cells, has shown good efficacy in an animal model [138]. Eganelisib (Infinity Pharmaceuticals, Cambridge, MA, USA), also known as IPI-549, a selective small molecule PI3Kγ inhibitor, could be used to reprogram the TAM to M1 macrophages, as well as to increase the tumor-infiltrating lymphocytes (TIL) that could promote CTL-mediated tumor regression without targeting cancer cells directly [138]. An interim analysis of an ongoing trial (NCT02637531) with IPI-549, as monotherapy and in combination with nivolumab, has reported favorable tolerability, early signs of clinical activity, and evidence of immune modulation [56]. This recently discovered strategy has already been granted the *Fast track* designation by FDA for use, in-combination with an immune checkpoint inhibitor and chemotherapy, as the first-line treatment of patients with inoperable locally advanced or metastatic triple-negative breast cancer, and in combination with nivolumab (Opdivo), for the treatment of patients with advanced urothelial carcinoma. It is currently being evaluated in HNSCC via a phase II window of opportunity trial (NCT03795610).

#### 3.4.4. Telomerase

Telomerase, an anti-apoptotic enzyme, is re-expressed in most tumor cells. It ensures the reconstitution of the telomeres that are shortened at each cell division, and thus prevents the entry of the cell into replicative senescence. This enzyme comprises a catalytic subunit called human telomerase reverse transcriptase (hTERT), and a structural subunit composed of a telomerase RNA component (TERC). The main mechanism of telomere maintenance in cancers depends on the reactivation of hTERT, which is overexpressed in almost all cancers offering them a form of immortality [139,140]. In fact, one of the mechanisms by which high-risk-HPV get involved in the process of carcinogenesis is by the promotion of hTERT, an activity mediated by E6 and E7 oncogenes of HPV [141,142]. A group of investigators from France has identified four novel major histocompatibility complex (MHC) class II–restricted peptides derived from hTERT as “*Universal Cancer Peptides*” (UCP), and have validated their ability to induce a tumor-specific immune response [143,144]. They developed a novel UCP-based anti-tumor vaccine called *UCPVax*, which is made up of two separate peptides called UCP2 and UCP4, derived from hTERT [143]. Based on these hypotheses and the backing evidence, and to further validate and expand the role of this vaccine, these investigators have initiated a multicentric, phase II trial (NCT03946358) called *VolATIL* (UCPVax Vaccine and Atezolizumab for the Treatment of HPV+ Cancers) in February 2020. The objective of this trial is to determine the clinical response and immunological efficacy of combining the UCPVax with atezolizumab, an anti-PD-L1, in HPV+ cancers, including HNSCC [144]. Intriguingly, another ongoing phase II trial (NCT05075122) called the *FOCUS study* is also investigating the tolerability and efficacy of another *universal cancer* vaccine (*UV1)-* in HNSCC. The novel UV1 vaccine consists of three SLPs representing 60 amino acids of the hTERT subunit of human telomerase. The UV1 has received the designation of *Fast track* from the FDA for its use in unresectable or metastatic melanoma as an adjuvant to the treatment with pembrolizumab or ipilimumab.

### 3.5. Co-Stimulation/Modulation of Anti-Tumor Immunity

#### 3.5.1. OX40 Agonists

PD-1 and CTLA-4, two well-known ICs, contribute to immunosuppression by preventing optimal T cell activation via various mechanisms. One such mechanism is TCR stimulation, which induces T cell exhaustion and apoptosis. OX40, a costimulatory molecule expressed transiently on the surface of T cells upon TCR activation, plays a key role in maintaining CD4^+^ and CD8^+^ T cell function by inducing their proliferation, differentiation, and survival. It is also known to decrease the immune-suppressive TME by significantly reducing the Treg and MDSC population, and TGF-β expression [145]. Preclinical studies have shown that the agonists of OX40 enhance the host’s anti-tumor immunity, either alone or in combination with ICI such as anti-PD-1, anti-PD-L1, and anti-CTLA-4 [145].

The ability of the OX40 agonist to generate potent anti-tumor immunity was clinically validated for the first time in a small cohort of advanced tumors (NCT01644968) [146]. Subsequently, a phase Ib trial (NCT02274155) demonstrated the safety and immunological activity of OX40 agonists in HNSCC. In this trial, apart from the increase in CD4+ and CD8+ T cell proliferation after vaccination, a comparison of tumor specimens before and after treatment revealed an increase in activated, conventional CD4+ TIL in most patients and higher clonality by TCRβ sequencing [57]. An OX40 agonistic humanized monoclonal antibody (MEDI0562) has also been shown to have a tolerable safety profile and measurable immune-related responses in a phase I trial (NCT02318394) on 55 patients with heavily pre-treated solid tumors (including 26 HNSCC) [147]. Currently, this molecule is being evaluated (NCT03336606) as a neoadjuvant prior to surgical resection of 35 patients with HNSCC or melanoma. On the other hand, a recently introduced recombinant humanized hexavalent OX40 agonist called INBRX-106 (Inhibrx, Inc., La Jolla, CA, USA) is also under evaluation for its safety profile and for the MTD and/or RP2D, both as a monotherapy and in combination with pembrolizumab (NCT04198766). Currently, the OX40 agonist is also being evaluated in combination with a personalized medicine strategy (NCT03739931), the details of which can be found in corresponding section of this manuscript. Overall, with the results of these ongoing trials, OX40 agonists could be amongst the prominent immunotherapeutics for HNSCC in future.

#### 3.5.2. TLR Agonists

TLR agonists are known for their role in modulating anti-tumor immunity and for their ability to induce pro-inflammatory cytokines [148]. Stimulation of TLRs induces natural killer (NK) cell activation, increases antibody-dependent cell-mediated cytotoxicity (ADCC), and induces Th1-polarizing cytokines [58]. A selective TLR8-agonist called moltolimod (APExBio, Houston, TX, USA), also known as VTX-2337, has demonstrated its ability to augment the clinical response of cetuximab (a clinically approved and EGFR-specific monoclonal antibody), in cases of LA or R/M HNSCC [58,59]. A large, randomized, placebo-controlled trial called the *Active8 study* (NCT01836029) compared the EXTREME regimen (one of the standard of care treatment for R/M HNSCC) in combination with motolimod or a placebo in 195 patients. Although the survival outcomes did not differ with the addition of motolimod in the overall cohort, there was a significant survival benefit in a sub-group of HPV-positive tumors [60].

Similar trials (NCT01360827 and NCT01040832) with a novel TLR9 agonist, called *EMD 1201081* (Aceragen Inc, Cambridge, MA, USA), also known as HYB-2055, IMO-2055 or IMOxine, have also failed to demonstrate incremental clinical efficacy when added to cetuximab in cetuximab-naïve patients with R/M SCCHN [61]. Another TLR9 agonist called CMP-001 (Checkmate Pharmaceuticals Inc, Cambridge, Massachusetts, USA), also known as vidutolimod, has successfully induced an anti-tumor T cell response via the production of IFN-α in an in vitro study [149]. In July 2020, the FDA granted *Fast track* status to CMP-001, in combination with nivolumab plus ipilimumab, for use as a first line therapy in unresectable advanced melanoma and for treatment of metastatic melanoma refractory to prior anti-PD-1 blockade. *CMP-001-007* is an ongoing multicenter phase II trial (NCT04633278) that has been evaluating the intratumoral CMP-001 in combination with pembrolizumab in 24 patients with R/M HNSCC since November 2020.

A group of researchers from the Netherlands worked on vaccines containing TLR-ligands (TLR-L) that covalently bound to antigenic SLP, and successfully demonstrated their capacity to induce the antigen-specific CD8+ and CD4+ T cell responses required for anti-tumor effects in preclinical studies [150,151]. Particularly, they developed a novel TLR2-L called Amplivant (AV)(ISA Pharmaceuticals, Leiden, Oegstgeest, The Netherlands) and reported a strong potency of AV-SLP conjugates in inducing DC maturation, in vivo T cell priming, and anti-tumor immunity, which correlated well with the therapeutic effect in a murine tumor model [151]. Inspired by the positive results of their preclinical studies, they designed a dose-escalation phase I vaccination trial (NCT02821494), a first-in-human trial, to test the safety and immunogenic potency of the AV-SLP vaccine. For this trial, the two SLPs derived from the two most immunodominant regions of the HPV16 E6 oncoprotein (E6 71–95 and E6 127–158) were conjugated to AV to produce a vaccine called *HESPeCTA* (HPV E Six Peptide Conjugated To Amplivant). Here, *HESPeCTA* was administered intradermally to 25 enrolled individuals, 16 with previously treated (currently disease-free) HPV16+ malignancy (12 OPSCC) and nine with HPV-16 associated premalignant lesions. This delivery mode was chosen to take advantage of the direct loading potency of skin-resident DCs with vaccines. The results of this study, published recently, reported an induction of a robust HPV16-specific T cell immunity with the intradermal AV-SLP vaccine in patients who had previously been treated for HPV-16 positive (pre-) malignancies [62]. In addition, the increased vaccine dose led to a higher number of mild adverse events, but a stronger systemic T cell immunity, and the responses were persistent until the end of the trial. Although further evaluation is essential for the safety and clinical application of the *HESPeCTA* by larger trials, the initial results are promising regarding its role as an adjuvant.

#### 3.5.3. IL

Agonists of several key cytokines involved in the proinflammatory cascade can be used for inducing or potentiating anti-tumor immunity in HNSCC.

##### IL-2

The role of IL-2 in enhancing anti-tumor activity is well established. As mentioned earlier, under the section of whole ATC vaccines, a combination of preconditioning with IL-2 prior to subsequent vaccination led to an augmented anti-tumor response in HNSCC patients [100,101]. Nevertheless, over the years, several IL-2-based molecules have been introduced and are being evaluated in HNSCC. *NKTR-214* (Nektar Therapeutics, San Francisco, California, USA and Bristol Myers Squibb, New York, NY, USA), also known as *Bempegaldesleukin*, is an immunostimulatory IL-cytokine designed to provide a controlled and sustained signal to the IL-2 receptor pathway. It is currently being evaluated in a phase II trial (NCT04936841) involving HNSCC. A*LKS 4230* (Alkermes, Inc., Dublin, Ireland), also known as *Nemvaleukin alfa*, is a fusion protein of circularly permuted IL-2 and the extracellular domain of CD25. It has shown good tolerance and acceptable anti-tumor effects with pembrolizumab in 14 patients of advanced and recurrent HNSCC in a phase II trial (NCT04144517) [63].

##### IL-15

*N-803* (ANKTIVA, ImmunityBio Inc., El Segundo, CA, USA), formerly known as ATL-803 or *Nogapendekin alfa*, is a novel IL-15 superagonist complex consisting of an IL-15 mutant (IL-15N72D) bound to an IL-15 receptor α/IgG1 Fc fusion protein. In a first-in-human trial (NCT01727076) of ALT-803, the vaccine, injected subcutaneously, was well tolerated, with minimal cytokine toxicities, and led to substantially increased circulating NK and CTL in LA solid tumors, including HNSCC [64,65]. ALT-803 is also known to rescue checkpoint activity in a checkpoint-independent manner via its selective enhancement of NK and CTL number and function, without stimulation of T-reg and MDSC [66]. It has also demonstrated its ability to potentiate the anti-tumor activity of cetuximab in EGFR-positive HNSCC in a mouse model [152]. *QUILT-3.055* is an active phase IIb multicohort study (NCT03228667) that is evaluating the combination of *N-803* with investigator choice ICI in 145 patients with 11 different types of advanced tumors, including HNSCC. Preliminary data analysis from the 135 patients of this trial has demonstrated a low toxicity profile and promising clinical efficacy, as well as a durable therapeutic response, in patients who had previously progressed on the same ICI [66]. *N-803* is also currently being evaluated in combination with other immunotherapeutic strategies, such as *chimeric antigen receptor (CAR)-T cell therapy* (NCT04847466) and Anti-PD-L1/TGF-beta ‘Trap’ with *Bintrafusp alfa* (M7824) *plus TriAd Vaccine* (ETBX-011, ETBX-051, and ETBX-061) (NCT04247282, a Sequential Window of Opportunity Trial), in HNSCC. Recently, when the present review was being compiled, the investigators of the latter trial published their results of monotherapy with *Bintrafusp alfa* in 14 patients of newly diagnosed HPV-unrelated HNSCC [153]. They reported the strategy of dual PD-L1 and TGF-β blockade to be safe and effective, as they noted grade III toxicity in one and grade IV in none of the patients, and at least a partial pathological response in the primary tumor or nodal disease in 43% of the patients [153]. Other Cytokine Stimulators

In a phase I/II trial (NCT01468896), recombinant IL-12, called *Edodekin alfa*, exhibited its safety profile in combination with cetuximab and demonstrated increased ADCC with greater production of IFNγ, IFNγ inducible protein (IP)-10, and TNF-α [68]. Interestingly, a recombinant human IL-7 called NT-I7 (NeoImmuneTech, NeoImmuneTech, Rockville, MD, USA), also known as *Efineptakin alfa*, is currently being evaluated for its safety and dosage in a window of opportunity trial (NCT04588038) on salvage surgery for recurrent HNSCC. IRX-2 (Brooklyn ImmunoTherapeutics, Brooklyn, NY, USA) is a complex proprietary therapeutic containing numerous active cytokine components that are supposed to reduce the immune suppression in TME and activate a coordinated immune response against the tumor. It showed its safety and efficacy as a neoadjuvant prior to the main treatment in 27 patients with treatment-naïve HNSCC in a phase II trial (NCT00210470) [67,154].

### 3.6. Personalized Medicine in HNSCC

#### 3.6.1. Neoantigen-Based Individualized Therapeutic Vaccines

Each of the malignant tumors exhibit numerous, but unique, sets of somatically mutated proteins, and such neoantigens can serve as targets for the development of individualized therapeutic vaccines, also known as *personalized cancer vaccines (PCV)* [155]. In other words, *PCV*s rely on the specific neoantigens derived from the patient’s tumor tissue itself to induce the ASIR against the tumor cells [156]. Powered by the advent of next-generation genomic sequencing technologies and computational algorithms, it is now possible to efficiently map the individual cancer’s *mutanome* (tumor-specific repertoire of immunogenic antigens), which ultimately aids in the selection of the most suitable target neoantigen for preparing the PCV [19,155]. The *SQZ-PBMC-HPV* vaccine, *Tabelecleucel* for EBV+ NPC, and ATC-based vaccination strategies discussed in the earlier sections are actually some of the examples of PCV that are under trial for safety and efficacy in HNSCC. Currently, various other platforms and technologies are also being explored to construct an efficient PCV for use in HNSCC, as listed below.

*AlloVax* (Immunovative Therapies Ltd., Jerusalem, Yerushalayim, Israel) is a PCV combining chaperone-rich cell lysate (CRCL) as a tumor antigen source prepared from a patient’s tumor and AlloStim™ as an adjuvant. AlloStim™ (Immunovative Therapies Ltd., Jerusalem, Yerushalayim, Israel) is a living, non-genetically manipulated, allogeneic cell therapy, that is purified, expanded and differentiated from the blood of normal donors. The therapy with Allostim™ is supposed to convert the cold tumors to hot and naturally down-regulate the IC in TME [157]. Nevertheless, in a phase II trial (NCT01998542) with ten patients of pretreated R/M HNSCC, the *AlloVax* vaccine was well-tolerated, with 50% of the patients showing a visible clinical response that correlated with the anti-tumor immune response [69]. A phase I/IIa study (NCT03633110) evaluated the safety, tolerability, immunogenicity, and anti-tumor activity of *GEN-009* (Genocea Biosciences, Inc., Cambridge, MA, USA), an adjuvanted PCV containing up to 20 neoantigens selected by ATLAS™ (Antigen Lead Acquisition System). ATLAS™ is the proprietary technology platform of the parent company that quickly identifies a few appropriate vaccine candidates from thousands of potential tumor antigens evaluated. In this trial, ATLAS was used to identify neoantigens in each patient’s tumor, recognized by their CD4 and/or CD8 T cells, and the identified neoantigens were incorporated into a patient’s personalized vaccine in the form of SLPs. The vaccines were given to 15 participants with advanced cancers (including HNSCC) as a combination therapy with an ICI and as a monotherapy in one other patient. Overall, the combination was well-tolerated and elicited robust and durable induction of broad neoantigen-specific immune responses [70]. Currently, two separate phase I trials are evaluating the safety and efficacy of two other PCVs, called *personalized neoantigen peptide-based vaccine*, also known as *PNeoVCA*, (NCT05269381) and *MVX-ONCO-1* (NCT02999646). In the former, the safety and tolerability of *PNeoVCA* (Pepscan, Lelystad, The Netherlands), a PCV containing a pool of 20 unique peptides, is being explored in combination with pembrolizumab. *MVX-ONCO-1* (MaxiVAX SA, Geneva, Switzerland) is a PCV consisting of irradiated, autologous tumor cells as the antigen sources and an immune-modulator (GM-CSF) released from an immuno-protected, encapsulated, allogeneic, genetically modified cell line (MVX-1). An earlier phase I trial (NCT02193503) of MVX-ONCO-1 in LA or R/M solid tumors, including HNSCC, stopped recruiting after 34 patients, but is still active. An interim analysis of 11 patients of HNSCC from both these trials of MVX-ONCO-1 showed the vaccine to be safe and effective in producing a prolonged clinical response in patients subsequently treated with nivolumab or cisplatin-based chemotherapy [71].

*QUILT-2.025* is a phase I trial (NCT03552718) that was conceived for the purpose of evaluating the safety, RP2D, and preliminary ASIR efficacy of a personalized *NANT neoepitope yeast-based vaccine* called *YE-NEO-001* (NantBioScience, Inc., Los Angeles, CA, USA) in patients with previously treated solid tumors, including HNSCC. In this trial, a personalized recombinant yeast-based vaccine was engineered to express multiple neoantigen epitopes (neoepitopes) based on an individual subject’s tumor molecular profile. However, the status of the trial, which began in Aug 2018, is currently not publicly known. On the other hand, another randomized Phase I trial (NCT04183166) is studying the *TG4050* (Transgene, Illkirch-Graffenstaden, France), an MVA-based therapeutic vaccine based on the *myvac*^TM^ platform, in patients with newly diagnosed LA HNSCC.

A first-in-human phase I/IIa study (NCT03548467), designed to evaluate the safety, feasibility, and efficacy of multiple dosing with individualized *VB10.NEO* (Nykode Therapeutics ASA, Oslo Research Park, Oslo, Norway) and *bempegaldesleukin* (NKTR-214, Nektar Therapeutics, San Francisco, CA, USA) immunotherapy in patients with LA or metastatic solid tumors, including HNSCC, is currently active. As per the update on ClinicalTrails.gov, VB10.NEO immunotherapy will commence as soon as the patient-specific VB10.NEO vaccine is available if the patient-specific vaccine meets all pre-specified product release criteria. Additionally, an ongoing phase I study (NCT03739931) is evaluating intratumoral injections of *mRNA-2752* (ModernaTX, Inc., Cambridge, MA, USA), a lipid nanoparticle encapsulating mRNAs encoding human OX40L, IL-23, and IL-36γ (pro-inflammatory cytokines), both as monotherapy and in combination with an ICI (durvalumab) in participants with R/M solid tumor malignancies or lymphoma, including HNSCC. An interim report of this trial with 23 subjects demonstrated its safety and sustained immunomodulatory effect [72]. Last but not least, another phase I clinical trial (NCT04266730) is planned to determine the safety of the *personalized and adjusted neoantigen peptide vaccine (PANDA-VAC)* administered concurrently with pembrolizumab in lung cancer and HNSCC. Therein, the investigators plan to perform whole exome and single-cell sequencing studies, using archival tumors and matched normal samples, to identify tumor-specific mutations and predict personalized HLA binding proteins. Based on this information, six neoantigens will be selected for inclusion in the primary PCV.

#### 3.6.2. Personalized T Cell Therapies

Adoptive cell therapy (ACT) is a form of personalized therapy in which a patient’s own immune cells are removed, expanded, or engineered in vitro, and then infused back to the patient to eliminate tumor cells [158]. T cells form the basis for several types of ACT, such as infusion of TIL, TCR-T, and CAR-T [158]. In fact, the ACT has made tremendous progress in recent times since the introduction of CAR-T, and recently, the FDA approved five CAR-T therapies for use in hematological malignancies of B-cell origin [159,160,161]. In ACT, either the tumor-specific T cells isolated from a patient (TIL) or those generated by genetic engineering of peripheral T cells from a patient or heathy donor (TCR-T and CAR-T), are expanded under optimal culture conditions and infused back to the same patient to recognize and target the tumor cells [158]. Theoretically, the typical advantages of adequately rewired T cells in immunotherapy include improved clinical action, which is attributable to increased T cell expansion, less T cell exhaustion, fewer chances of immune escape, and limited toxicity owing to their affinity towards tumor-specific antigens [158].

The ACT, in the form of TIL, has shown long-standing promising results in other tumors, but its efficacy is yet to be established in HNSCC [162]. On the other hand, TCR-T and CAR-T therapies are being explored by several authors in HNSCC, and the preliminary results are encouraging, both in terms of safety and clinical efficacy [163]. The isolated peripheral T cells can be genetically engineered to target several known *tumor antigens* of HNSCC, including, EGFR, MAGE-A4, MUC1, CD 70, and HER2, and the ACT with these agents has demonstrated a durable clinical response in early clinical studies [163,164,165]. Interestingly, an ongoing phase II trial (NCT04847466) is evaluating the effectiveness of irradiated PD-L1 CAR-NK cells in subjects with R/M gastric cancer or HNSCC in combination with pembrolizumab and N-803. Despite the unprecedented clinical responses to the ACT, even in patients with otherwise refractory tumors, there are several roadblocks to this approach. These obstacles are currently being worked around to ensure the smooth translation of this promising strategy from the lab to the bedside [158,160].

## 4. Future Perspectives

While there is no doubt about the eventual approval and wide-spread clinical applications of the therapeutic vaccines in HNSCC, in order to *Fast track* their case, some of the key challenges must be addressed as early as possible. The most critical of these include the predominance of an immune-depleted state in the majority of the HNSCC cases, rendering several of the existing vaccination strategies ineffective, and on the other hand, the technical limitations as well as financial constraints involved in the production of vaccines [166]. While the approaches to convert the immunophenotypically *cold* tumors into *hot* before vaccination, and the use of vaccines in combination with other immunotherapeutic methods such as ICI are some of the mechanisms to address the former, there is still a need for further research to enhance the cost-effectiveness of vaccine production. Studies and strategies to expand therapeutic vaccination from its present state of mostly adjuvant or palliative for R/M HNSCC to other clinical indications are also essential. The application of nanotechnology in the development of therapeutic vaccines, which has been undertaken in recent years, is expected to address some of the production- and efficacy-related concerns of the therapeutic vaccines for HNSCC [163].

## 5. Conclusions

The popularity of therapeutic vaccination has been advancing rapidly in HNSCC over the last few years. Some of these strategies are just a few steps away from becoming clinical realities. The major tumor antigens that have been explored as targets for designing therapeutic vaccines include HPV-16/-18 E6 and E7, MAGE, p53, EGFR, MUC1, and hTERT. Apart from targeting the specific tumor antigens expressed by the tumor cells, reprogramming the TME to potentiate intrinsic anti-tumor immunity and PCVs aimed at the individualization of therapeutic vaccines are some of the exciting emerging strategies. Particularly, prospects and tools *to transform the “cold tumors” into “hot”* have rejuvenated interest among clinicians and researchers alike. Amongst all the explored candidate vaccines in HNSCC, the likes of ISA101b, PDS0101, UV1, Tadalafil, OX40 agonists, N-803, and a battery of PSVs have demonstrated promising outlooks and are among the frontrunners for obtaining FDA approval for their clinical use in these tumors. However, most of the emerging evidence supports the role of these vaccines in HNSCC as adjuvants to other established regimens, such as ICI or chemoradiation, primarily in LA or R/M setting. Further large pre-clinical and clinical studies are needed to validate the other roles of these therapeutic vaccination strategies.

## Figures and Tables

**Table 1:** Various platforms of therapeutic vaccine delivery.

**Platforms**	**Peculiar Characteristics**
Autologous tumor cell vaccines	Prepared using patient-derived tumor cells, typically irradiated and combined with an immunostimulatory adjuvant, then administered to the same individual Entire spectrum of TAA is presented to patient’s immune system Preparation requires adequate tumor specimen, thus is difficult to manufacture
Allogenic whole tumor cell vaccine	Contain two or three established human tumor cell lines Limitless sources of tumor antigens Standardized and large-scale vaccine production is possible, thus could be cost-effective
DC vaccines	Patients’ autologous DCs are loaded with TAA and fused to adjuvants. DC, being the most potent APC, can co-stimulate the anti-tumor immunity when given with other active immunotherapeutic. Require leukaphereses to isolate peripheral blood mononuclear cells from patient Require cell culture processing, thus limiting the number of vaccinations
Peptide vaccines	Deliver the MHC class I restricted peptide epitopes, derived from TAA Simple to produce, safe, and booster dose-feasible Usually water-soluble and could be freeze-dried, but is stable at room temperature; thus, easy for storage and distribution, and cost-effective Free of bacterial/viral contaminating substances and devoid of oncogenic potential Prone to developing tolerance Rapidly degraded by serum/tissue peptidases, thus immune responses may be transient and/or of low magnitude Often need to be combined with immunogenic adjuvants
DNA vaccines	Ability to incorporate multiple genes into the vector Can modulate intracellular routing and modification of antigens as well as subsequent immune outcomes Can be rationally combined with other immunostimulatory agents, such as TLR agonists, to optimize antibody responses Modest efficacy Risk of genetic recombination, leading to reduced efficacy
RNA vaccines	Together with other agents for stabilization or adjuvant effects, such as liposomes or protamines Less likely to cause side effects or autoimmune diseases due to their rapid degradation and clearance
Viral vaccines	Viral vectors with low disease-causing potential and low intrinsic immunogenicity are engineered to encode TAAs with or without immunomodulating molecules Known to induce specific immune responses, both humoral and cell-mediated

Abbreviations (in alphabetical order): APC—Antigen presenting cell; DC—Dendritic cell; DNA—Deoxyribonucleic acid; MHC—Major histocompatibility complex; RNA—Ribonucleic acid; TAA—tumor associated antigen; TLR—Toll like receptors.

**Table 2:** Detailed list of major trials related to therapeutic vaccines for HNSCC.

**Sl No**	**Vaccine Name**	**Constituents**	**Platform**	**Trial Number**	**Initiated**	**Phase**	**Adjuvant or Combination**	**Patient Profile**	**Enrolment**	**Status**
1. Targeting HPV associated antigens										
1	*ISA 101b*	SLP derived from HPV-16 E6 and E7	Peptide	NCT02426892	Dec 2015	II	Nivolumab	incurable HPV-16+ OPSSC	22/24 *	Completed [41]
				NCT03258008	Apr 2018	II	Utomilumab	R/M/P checkpoint naïve HPV+ OPSCC	3/27	Terminated
				NCT03669718	Nov 2018	II	Cemiplimab	HPV-16+ R/M OPSCC	194	Recruiting
				NCT04369937	Jul 2020	II	Pembrolizumab + Cisplatin-based chemoradiotherapy	Treatment naïve HPV-16+ LA HNSCC	50 (e)	Recruiting
				NCT04398524	Jul 2021	II	Cemiplimab	HPV-16+ R/M OPSCC	86 (e)	Recruiting
2	*INO-3112*	DNA plasmid against HPV-16 and -18 E6 and E7 antigens	DNA	NCT02163057	Aug 2014	I/IIa	(IL-12)	HPV-16/-18 + HNSCC	22	Completed [42]
				NCT03162224	Jun 2017	Ib/IIa	Durvalumab	R/M HPV-16/-18 + HNSCC	35/50	Terminated
				NCT04001413	Sep 2019	II	Durvalumab	HPV 16+ OPSCC	0	Withdrawn
3	*TG 4001*	MVA -express HPV-16 E6 and E7 + cytokine, IL-2	Viral vector	NCT03260023	Sep 2017	Ib/II	Avelumab	HPV-16 + R/M cancers, including HNSCC	150 (e)	Recruiting (interim report) [43]
4	*HB-201* and *HB-202*	TheraT vector(s) expressing HPV16 E7E6	Viral vector	NCT04180215	Dec 2019	I/II	Pembrolizumab	HPV-16+ R/M cancers including HNSCC	200 (e)	Recruiting (interim report) [44]
5	*ADXS 11-001*	Attenuated Lm -LLO, engineered to secrete an HPV-E7 tumor antigen	Bacterial vector	NCT01598792	Feb 2012	I	-	HPV-16+ OPSCC	2	Terminated
				NCT02002182	Dec 2013	WOT	Neoadjuvant vaccine before TORS	HPV+ OPSCC	15/30	Completed (interim report) [45]
				NCT02291055	Apr 2015	I/II	Durvalumab	LA/M cervical cancers or HPV + HNSCC	66 (e)	Unknown
6	*PDS0101*	Liposomal-based HPV-16 E6/E7 multipeptide vaccine	Peptide (nano-particle based)	NCT04287868	Jun 2020	I/II	NHS-IL12 + Bintrafusp alfa	LA/M HPV+ Cancers including OPSCC	51	Active, not recruiting
				NCT04260126	Mar 2021	II	Pembrolizumab	R/M HPV-16+ HNSCC	95 (e)	Recruiting
				NCT05232851	Mar 2022	I/II	Pembrolizumab	HPV+ LA OPSCC	24 (e)	Recruiting
7	*SQZ-PBMC-HPV*	Generated from PBMC squeezed with HPV16 E6 and E7 antigens	Autologous	NCT04084951	Jan 2020	I	Atezolizumab or Other ICI	HPV16+ LA or R/M Solid Tumors	200 (e)	Recruiting (interim report) [46]
8	*DPX-E7*	HPV16-E711-19 nanomer	Peptide	NCT02865135	Dec 2016	Ib/II	-	R/M HPV-16+ HNSCC, cervical Ca, anal Ca	11	Active, not recruiting
9	*PepCan*	Four synthetic peptides covering HPV16E6	Peptide	NCT03821272	Nov 2019	I/II	Candin	Previously treated HNSCC patients who are in remission	20 (e)	Recruiting
10	*CUE-101*	HPV16 E7 peptide epitope (E7_11–20_) +IL-2	Peptide	NCT03978689	Jul 2019	I	Pembrolizumab	HPV16+ R/M HNSCC	85 (e)	Recruiting (interim results) [47]
11	*HARE-40*	*HPV Anti-CD40 RNA Vaccine*	RNA	NCT03418480	Apr 2017	I/II	-	Previously treated disease-free HPV16+ cancers	44 (e)	Recruiting
12	*p16_37-63 peptide vaccine*	27-amino-acid-long p16(INK4a)-based peptide vaccine	Peptide	NCT01462838	Aug 2011	I/II	Montanide ISA-51	R/M HPV+ cancers including HNSCC	26	Prematurely terminated [48]
				NCT02526316	Jun 2015	I	Montanide ISA-51 + Cisplatin based chemotherapy +/-radiotherapy	HPV+ cancers including HNSCC	11	Completed
2. Targeting non-viral tumor antigens										
13	*Trojan vaccines*	MAGE-A3 or HPV-16 derived peptides	Peptide	NCT00257738	Nov 2005	I	Montanide ISA 51 and GM-CSF	P/R/M HNSCC	16/90 *	Completed [49]
14	*p53-specific autologous DC -based vaccine*		Peptide	NCT00404339	Sep 2005	I	DC	LA HNSCC after treatment	16/50 *	Completed [50,51]
15	*p53MVA vaccine*		Viral vector	NCT02432963	Jun 2016	I	Pembrolizumab	LA or R/M Solid cancers	11/19 *	Active, not recruiting [52]
16	*CIMAvax*	Recombinant human EGF-rP64K	Peptide	NCT02955290	Dec 2016	I/II	Montanide ISA 51 + Nivolumab	Metastatic NSCLC or HNSCC	193 (e)	Recruiting
3. Targeting TME										
17	*Tadalafil*	PDE-5 inhibitor		NCT00894413	May 2007	II	-	Newly diagnosed or recurrent HNSCC	45	Completed [53]
				NCT00843635	Sep 2008	I	Surgery	OSCC or OPSCC undergoing Surgery	35	Completed [54]
				NCT01697800	Sep 2012	II	Conventional therapy	Newly diagnosed or recurrent HNSCC	40	Completed
				NCT02544880	Apr 2016	I/II	Anti-MUC1	Resectable recurrent or second primary HNSCC	14/16	Completed [55]
18	*UCPVax*	Universal cancer peptides derived from hTERT		NCT03946358	Feb 2020	II	Atezolizumab	HPV+ cancers including HNSCC	47 (e)	Recruiting
19	*UV1*	Three SLPs from hTERT		NCT05075122	Aug 2021	II	Sargramostim	R/M PDL1+ HSNCC	75 (e)	Recruiting
20	*IPI-549*	A specific PI3Kγ inhibitor		NCT02637531	Dec 2015	I/Ib	Nivolumab	LA or metastatic solid tumors, including HNSCC	219	Active, not recruiting (interim report) [56]
				NCT03795610	Mar 2020	II	Surgery	LAHNSCC undergoing surgical excision	15 (e)	Recruiting
4. Co-stimulation strategies										
21	*MEDI6469*	Murine anti-human OX40 agonist antibody		NCT02274155	Oct 2014	Ib	Surgery	Surgically resectable LA HNSCC	17	Completed [57]
	*MEDI0562*	Humanized OX40 agonist		NCT02318394	Mar 2015	I	-	Heavily pre-treated solid tumors, including HNSCC	55	Completed
				NCT03336606	Jul 2018	Ib	Surgery	Surgically resectable HNSCC or melanoma	35 (e)	Active, not recruiting
	*INBRX-106*	Hexavalent OX40 agonist antibody		NCT04198766	Dec 2019	I	Pembrolizumab	LA or metastatic solid tumors, including HNSCC	200 (e)	Recruiting
22	*VTX-2337*	TLR8-agonist		NCT01334177	Jun 2011	I	Cetuximab	LA or R/M HNSCC	13	Completed [58,59]
				NCT01836029	Oct 2013	II	EXTREME regimen	R/M HNSCC	195	Completed [60]
23	*IMO-2055*	TLR 9-agonist		NCT01040832	Dec 2009	II	Cetuximab	Cetuximab-naïve subjects with R/M HNSCC	107	Completed [61]
				NCT01360827	Aug 2010	Ib	5-FU/Cisplatin and Cetuximab	R/M HNSCC	13	Terminated
24	*CMP-001*	VLP-containing TLR 9 agonist		NCT04633278	Nov 2020	II	Pembrolizumab	R/M HNSCC	24	Active, not recruiting
25	*HESPeCTA*	HPV E six peptides conjugated to amplivant	SLP	NCT02821494	Mar 2015	I	-	HPV+ tumors or premalignant conditions	25	Completed [62]
26	*NKTR-214*	IL-2 agonist	Protein	NCT04052204	Dec 2019	Ib/II	Avelumab plus Talazoparib or Enzalutamide	LA or R/M HNSCC and mCRPC	3	Terminated
				NCT04936841	Aug 2021	II	Radiotherapy and pembrolizumab	R/M HNSCC	5	Active, not recruiting
27	*ALKS 4230*	IL-2 and extracellular domain of CD25		NCT04144517	Feb 2020	II	Pembrolizumab	Advanced or recurrent HNSCC	14	Completed [63]
28	*ALT-803*	Recombinant human IL15		NCT01727076	Feb 2013	I	-	LA or recurrent solid tumors, including HNSCC	20	Completed [64,65]
29	*N 803*	IL-15 superagonist complex		NCT03228667	Dec 2018	IIb	ICI	R/M Solid tumors including HNSCC	135/145 *	Active, not recruiting (Interim report) [66]
30	*Irradiated PD-L1 CAR-NK cells*	Autologous CAR-T cell therapy		NCT04847466	Dec 2021	II	N-803 + Pembrolizumab	R/M gastric or HNSCC	55 (e)	Recruiting
31	*M7824*	Anti-PD-L1/TGF-beta Trap		NCT04247282	Jun 2020	WOT	TriAd Vaccine + N-803	HNSCC	21	Active, not recruiting
32	*IRX-2*	Numerous active cytokine components		NCT00210470	Jul 2005	II	Cyclophosphamide, indomethacin, and zinc	Treatment-naïve HNSCC	27	Completed [67]
33	*Edodekin alfa*	Recombinant interleukin-12		NCT01468896	Oct 2011	I/II	Cetuximab	Unresectable primary or recurrent HNSCC	23	Completed [68]
34	*NT-I7*	Recombinant human IL-7		NCT04588038	Mar 2021	WOT	Surgery	Recurrent HNSCC undergoing salvage surgery	10 (e)	Recruiting
5. Personalized vaccines										
35	*AlloVax*	Chaperone-rich cell lysate		NCT01998542	Jan 2016	I	AlloStim	R/M HNSCC	10/12 *	Completed [69]
36	*GEN-009*	Up to 20 neoantigens		NCT03633110	Aug 2018	I/IIa	Nivolumab or Pembrolizumab	Solid tumors, including HNSCC	15/24 *	Completed [70]
37	*PNeoVCA*	Personalized neoantigen peptide-based vaccine		NCT05269381	Mar 2022	I	Sargramostim plus pembrolizumab	LA and R/M solid tumors, including HNSCC	36 (e)	Recruiting
38	*MVX-ONCO-1*	Irradiated, autologous tumor cells		NCT02193503	Mar 2014	I	GM-CSF	LA or R/M solid tumors, including HNSCC	34	Active, not recruiting
				NCT02999646	Jul 2018	II	GM-CSF	LA or R/M HNSCC	21 (e)	Recruiting (Interim report) [71]
39	*YE-NEO-001*	NANT neoepitope yeast-based vaccine		NCT03552718	Aug 2018	I	-	Previously treated solid tumors, including HNSCC	16 (e)	Unknown
40	*TG4050*	MVA based on the *myvac*^®^ platform		NCT04183166	Dec 2019	I	-	Treatment naïve LA HNSCC	30 (e)	Recruiting
41	*VB10.NEO*	DNA plasmid vaccine with intrinsic adjuvant effect		NCT03548467	Apr 2018	I/IIa	NKTR-214	LA or metastatic solid tumors, including HNSCC	65 (e)	Active not recruiting
42	*mRNA-2752*	Lipid nanoparticle encapsulating mRNAs encoding human OX40L, IL-23, and IL-36γ		NCT03739931	Nov 2018	I	Durvalumab	R/M solid tumors or lymphoma, including HNSCC	264 (e)	Recruiting (Interim report) [72]
43	*PANDA-VAC*	Personalized and adjusted neoantigen peptide vaccine		NCT04266730	Feb 2023	I	-	Advanced lung cancer and HNSCC	6 (e)	Not yet recruiting

Under the enrolment column, (e) = estimated enrolment and * = actual/estimated. Abbreviations (in alphabetical order): 5-FU—5-Flurouracil; CAR-T—chimeric antigen receptor T cells; DC—dendritic cell; DNA—deoxyribonucleic acid; EGF—epidermal growth factor; GM-CSF—granulocyte–macrophage colony-stimulating factor (sargramostim—recombinant GM-CSF); HNSCC—head and neck squamous cell carcinoma; HPV—human papilloma virus; hTERT—human telomerase reverse transcriptase; ICI—immune checkpoint inhibitor; IL—interleukin; LA—locally advanced; LA/M—locally advanced/metastatic; Lm-LLO—listeria monocytogenes listeriolysin O; MAGE—melanoma antigen-encoding gene; mCRPC—metastatic castration-resistant prostate cancer; MVA—modified vaccinia strain Ankara; OPSCC—oropharyngeal squamous cell carcinoma; OSCC—oral squamous cell carcinoma; P/R/M—progressive/recurrent/metastatic; PBMC—peripheral blood mononuclear cell; PDE-5—phosphodiesterase-5; PDL1—programmed cell death protein ligand1; R/M—recurrent/metastatic; RNA—ribonucleic acid; SLP—synthetic long peptides; TGF—transforming growth factor; TLR—toll-like receptor; TORS—transoral robotic surgery; VLP—virus-like particle; WOT—window of opportunity trial.

## Data Availability

No new data were created in this review.
